# Ocular benzalkonium chloride exposure: problems and solutions

**DOI:** 10.1038/s41433-021-01668-x

**Published:** 2021-07-14

**Authors:** Michael H. Goldstein, Fabiana Q. Silva, Nysha Blender, Trung Tran, Srilatha Vantipalli

**Affiliations:** Ocular Therapeutix, Inc, Bedford, Massachusetts USA

**Keywords:** Adverse effects, Pharmaceutics

## Abstract

Preservatives in multidose formulations of topical ophthalmic medications are crucial for maintaining sterility but can be toxic to the ocular surface. Benzalkonium chloride (BAK)—used in approximately 70% of ophthalmic formulations—is well known to cause cytotoxic damage to conjunctival and corneal epithelial cells, resulting in signs and symptoms of ocular surface disease (OSD) including ocular surface staining, increased tear break-up time, and higher OSD symptom scores. These adverse effects are more problematic with chronic exposure, as in lifetime therapy for glaucoma, but can also manifest after exposure as brief as seven days. Multiple strategies are available to minimize or eliminate BAK exposure, among them alternative preservatives, preservative-free formulations including sustained release drug delivery platforms, and non-pharmacological therapies for common eye diseases and conditions. In this paper, we review the cytotoxic and clinical effects of BAK on the ocular surface and discuss existing and emerging options for ocular disease management that can minimize or eliminate BAK exposure.

## 摘要

眼科外用药物多剂量配方中的防腐剂对维持药物无菌至关重要, 但其对眼表有毒性作用。苯扎氯铵 (BAK) 用于约70%的眼科药物中, 众所周知会对结膜和角膜上皮细胞造成细胞毒性损伤, 并引起眼表疾病 (OSD) 相关的症状和体征, 包括眼表染色、泪膜破裂时间延长和OSD症状评分升高等。这些不良反应在长期使用时更加严重, 如青光眼的终生治疗, 但有些患者在使用7天后也会表现出来。有多种策略可用于减少或消除BAK的不良反应, 其中包括更换防腐剂、使用无防腐剂的配方 (包括缓释给药平台) 以及使用常见眼病和眼部症状的非药物疗法等。在这篇文章中, 我们回顾了BAK对眼表的细胞毒性和临床影响, 并讨论了现有的和正在研究的可减少或消除BAK不良反应的眼病治疗方案。

## Preservatives used in eye drops

Preservatives serve a critical role in the formulation of topical ophthalmic medications used to treat a wide variety of ocular conditions. Required in multidose formulations by most regulatory bodies since the 1970s [1], their primary role is to provide antimicrobial activity to maintain sterility, thus cost-effectively extending shelf life [2]. Their effects, however, are not limited to infectious microbes—many preservatives can also cause significant damage to ocular tissues, particularly in the setting of chronic exposure.

Many different molecules have antimicrobial properties and have been incorporated into ophthalmic formulations. These have been extensively reviewed [2–7] and are summarized in Table 1. Of these, benzalkonium chloride (BAK) is the most commonly used and the most robustly studied. Several newer proprietary preservation systems—such as Polyquad, Purite, and SofZia—have been developed to mitigate undesirable attributes of BAK; these are particularly useful in glaucoma medications given the chronic polytherapy nature of glaucoma and the resulting extensive preservative exposure. Various others of these preservative classes have both intrinsic antibacterial and antifungal activity and are commonly used in contact lens solutions, artificial tears, and rewetting drops to reduce the risk of contact lens-associated fungal keratitis.

**Table 1:** Classes and representative molecules of common preservatives in ocular therapeutic formulations [2–7].

Chemical class	Component(s)	Proprietary name (if applicable)	Commercial formulation examples
Quaternary ammoniums (detergents)	Benzalkonium chloride		Many
	Polyquaternium-1	Polyquad (Alcon)	Travoprost PQ
	Polyquaternium-42		–
	Cetrimide		Some artificial tears
Oxidizing agents	Sodium perborate	GenAqua (Novartis), Dequest (TheraTears)	Some artificial tears
	Stabilized Oxochloro Complex (SOC)	Purite (Allergan, an AbbVie company), OcuPure (AMO)	Brimonidine P
Ionic buffers	Borate, sorbitol, propylene glycol, and zinc	SofZia (Alcon)	Travoprost Z
Amidines	Chlorhexidine		Some contact lens solutions
Parabens	Methylparaben		Some artificial tears
	Propylparaben		
Alcohols	Chlorobutanol		Some artificial tears
	Phenylethanol		
Mercury-based	Thimerosal		Pilocarpine
	Phenylmercuric nitrate/acetate		

Of these many molecules, Benzalkonium chloride (BAK) is used in approximately 70% of ophthalmic formulations [8, 9] and is thus the focus of this paper. BAK is a quaternary ammonium compound with both hydrophilic and hydrophobic elements that renders it highly hydrosoluble [3, 8]. Bactericidal activity occurs via interactions of BAK with bacterial cell membranes, leading to membrane instability and cell lysis [8]. BAK is effective against both Gram-positive and Gram-negative bacteria as well as fungi [3]. BAK may also act as a corneal penetration enhancer, leading to great ocular penetrance of active ingredients in BAK-preserved formulations [3, 10, 11].

The monograph aims to review the adverse effects of acute and chronic exposure to BAK and to describe options for reducing or eliminating exposure to BAK and other preservatives in topical ophthalmic medical therapy.

## Cytotoxic effects of BAK on ocular tissues

The cytotoxic effects of BAK on ocular tissue cells have been extensively documented (Table 2). The threshold concentration at which toxicity occurs has been estimated to be ~0.005%; as a preservative in topical ophthalmic formulations, BAK is commonly used in concentrations of 0.04–0.02% [3]. In both tissue culture and in animal models, BAK has been shown to reduce the survival of corneal [12–18], conjunctival [12, 13], trabecular meshwork (TM) [19, 20], and ciliary epithelial [19] cells. In animal models, BAK caused injury to corneal epithelial cells [15, 21, 22], loss of conjunctival goblet cells [22, 23], and delayed corneal wound healing [24]. BAK also induces lymphocyte infiltration of conjunctival epithelium and stroma in animal models [21, 22] and increases the levels of inflammatory markers in ocular tissues [15, 16, 18]. DNA fragmentation and oxidative damage have been demonstrated in TM cells exposed to BAK, leading to altered gene expression in these cells [20]. Further, BAK exposure induces corneal epithelial cell apoptosis [18]. Clinical studies suggest that these effects may be at least partially reversible upon withdrawal of BAK exposure [25–30].

**Table 2:** Cytotoxic effects of BAK on ocular tissues.

Decreased corneal, [12–18] conjunctival, [12, 13] trabecular meshwork, [19, 20] and ciliary epithelial cell [19] survival
Corneal epithelial cell injury [15, 21, 22]
Conjunctival goblet cell loss [22, 23]
Delayed corneal wound healing [24]
Lymphocyte infiltration of conjunctival epithelium and stroma [21, 22]
Elevates inflammatory marker concentrations in ocular tissues [15, 16, 18]
DNA fragmentation and oxidative damage in TM cells leading to altered gene expression [20]
Induction of corneal epithelial cell apoptosis [18]

The underlying mechanism(s) by which BAK damages cells of the ocular tissues has not been fully elucidated. BAK is a quaternary ammonium compound and is thus cationic, so it is possible that BAK interacts with mitochondria, which are the only negatively charged intracellular compartment. Short-term exposure to cultured conjunctival epithelial cells stimulates the production of hydrogen peroxide, a reactive oxygen molecule, resulting in partial mitochondrial dysfunction [31]. BAK has also been shown to trigger oxidative stress in mitochondria and mitochondrial fragmentation [32], and to inhibit mitochondrial function by >90% [33]. Mitochondrial oxidative stress is known to play a role in the development of ocular surface disease [34] as well as age-related corneal diseases and normal corneal aging [35].

## Clinical impact of BAK in topical ophthalmic therapeutics

Most of what it is known about chronic BAK exposure comes from patients with glaucoma receiving chronic therapy with one or more BAK-preserved medications dosed one or more times daily. Given the known cytotoxic effects of BAK on the ocular surface, one would expect a higher rate of ocular surface disease (OSD) in eyes with chronic BAK exposure. In fact, the prevalence of OSD among glaucoma patients has been reported to be 30–70% in various studies [36–42], which is much higher than in the 5–30% prevalence among similarly-aged adults without glaucoma [43]. This higher than expected rate of OSD in glaucoma patients has been linked to BAK in topical glaucoma medications [1–4, 6, 44]. The total BAK “dose” (number of medications, number of drops per day, duration of therapy, etc.) correlates with OSD prevalence and severity in glaucoma patients [36–42]. Clinical manifestations of BAK-induced ocular surface toxicity in glaucoma patients using BAK-preserved medications (Table 3) include pain and discomfort (including stinging, burning, foreign body sensation, itching, and ocular dryness) [45, 46], tearing [46], increased staining of conjunctival and corneal epithelial surfaces [27, 46–48], increased tear break-up time [25, 27, 30, 46, 47, 49–51], lower Schirmer scores [46, 47], higher prevalence of punctate keratitis [25, 26, 30], and overall worse scores on the Ocular Surface Disease Index (OSDI), a validated instrument for assessing the presence and severity of ocular surface symptoms [27, 30, 48, 49, 52, 53]. Other manifestations of chronic BAK exposure in eyes with glaucoma include conjunctival subepithelial inflammation and fibrosis [9, 54–56], which can reduce the success of subsequent filtering surgery [9, 57–59], as well as a higher rate of cataract surgery in eyes on long-term glaucoma therapy compared to those without such exposure [60, 61], potentially related to the known actions of BAK in increasing expression of inflammatory and apoptotic mediators in lens epithelial cells [62]. OSD as a comorbidity to glaucoma adversely affects the quality of life [63, 64], and the use of BAK-preserved medications has also been associated with worse quality of life than BAK-free medications [47, 52]. Further, interactions between the two conditions can make both harder to manage [65].

**Table 3:** Clinical manifestations of BAK toxicity.

Pain/discomfort [45, 46]
Tearing [46]
Ocular surface staining [27, 46–48]
Increased tear break-up time [25, 27, 30, 46, 47, 49–51]
Lower Schirmer scores [46, 47]
Punctate keratitis [25, 26, 30]
Worse OSDI scores [27, 30, 48, 49, 52, 53]
Subconjunctival inflammation/fibrosis [9, 54–56]
Reduced success of glaucoma filtering surgery, [9, 57–59]
Increased risk of cataract surgery [60, 61]
Reduced quality of life [47, 52]

Additional insight in BAK toxicity can be drawn from patients with OSD and allergy. OSD therapies are also formulated with preservatives, including BAK [66], which are known to cause or exacerbate OSD [67]. Topical anti-allergy medications preserved with BAK have also been shown to cause or aggravate OSD [44, 67], producing similar effects on the tissues of the ocular surface as were described for glaucoma and OSD above. Among patients with glaucoma and allergic conjunctivitis, a short 14-day course of BAK-preserved latanoprost increased eosinophil counts, altered results of the tear ferning test, and induced abnormalities in impression cytology [68], and even very brief exposure (one week) to BAK can induce goblet cell loss and induce other signs of OSD in subjects without preexisting OSD [69].

Also, BAK-preserved artificial tears alter the bacterial flora of the nasal mucosa reducing the population of *Staphylococcus epidermidis* [70], which could facilitate colonization with *S. aureus* species including pathogenic methicillin-resistant *S. aureus* [71, 72] as well as the influenza virus [73]. The effects of BAK on the novel human coronavirus SARS-CoV-2—the agent responsible for the COVID-19 pandemic—are unknown, although as a disinfectant, BAK is less effective than other biocidal agents in eradicating the virus from inanimate surfaces [74].

The diagnosis of BAK toxicity is clinical in nature. The clinical manifestations of BAK toxicity occur in a spectrum from subclinical to severe and are often overlooked. The onset can be insidious and appear weeks or months after starting therapy, or only after a threshold number of medications or drops per day is reached, confounding the causal relationship to therapy. Mild signs may be overlooked by busy clinicians who either do not recognize the findings or choose not to address them in non-complaining patients. Likewise, patients may not report symptoms, particularly if therapy is achieving therapeutic efficacy goals, choosing instead to accept redness, discomfort, or even blurred vision as a price to pay for satisfactory glaucoma control. For patients who do report bothersome symptoms, the well-described disconnect between the signs and symptoms of ocular surface disease (OSD) can result in a lack of objective findings [75, 76], failure to make the diagnosis of BAK-mediated OSD, and a missed opportunity to adjust therapy and preserve or improve quality of life.

## Approaches to minimize or eliminate BAK toxicity

Given the frequency and severity of BAK-related ocular toxicity, it is worthwhile to review the therapeutic options that can ameliorate BAK toxicity or reduce or eliminate BAK exposure in patients requiring short-term or long-term topical ophthalmic medical therapy (Table 4).

**Table 4:** Strategies for reducing or eliminating BAK exposure.

Fixed combinations to reduce daily drop frequency
Co-administration of ameliorators of BAK toxicity
Use of medications preserved with alternatives to BAK
Use of non-preserved medications
Use of sustained drug delivery formulations
Non-medication therapy

The adverse effects of BAK can be ameliorated by adding preservative-free lubrication formulated with taurine to the BAK-preserved medical regimen, which has been shown to improve tear break-up time (TBUT), conjunctival goblet cell density, and Ocular Surface Disease Index (OSDI) and Glaucoma Symptom Scale (GSS) scores [77]. For patients using multiple BAK-formulated products—a common scenario in glaucoma management—switching to a fixed combination product can reduce BAK exposure by reducing the total number of drops administered per day [64].

BAK exposure can be eliminated by using therapies formulated with preservatives other than BAK, by using preservative-free formulations (including emerging sustained drug delivery platforms), or by utilizing non-medical therapies (Table 4). Each of these options is discussed more fully below.

### Non-BAK preserved formulations

A number of alternative preservatives have been developed for topical ophthalmic therapeutics that are collectively less harsh and better tolerated than BAK. The key drawback to these preservatives is that they are proprietary in nature and are thus limited to use—at least currently—in formulations produced by their manufacturer.

#### SofZia

SofZia (Alcon Laboratories, Fort Worth, TX) is an ionic buffered solution containing borate, sorbitol, propylene glycol and zinc [66] that creates an oxidizing antimicrobial milieu in multidose bottles that meets US Pharmacopeia (but not European Pharmacopoeia) standards [78]. The components degrade quickly upon contact with cations on the ocular surface [79] resulting in a preservation system that is less cytotoxic than BAK to ocular tissues [12–14, 19, 21, 23, 24]. In clinical studies, travoprost preserved with SofZia yielded better scores on surveys of ocular symptoms and quality of life compared to BAK-preserved travoprost [52]. Also, in eyes switched from BAK-preserved to SofZia-preserved travoprost, significant improvements were observed in both ocular signs and symptoms of OSD [25–27]. Importantly in the context of BAK’s potential role in enhancing the penetration of active ingredients, there is no compromise of IOP control when BAK is replaced with SofZia in glaucoma medications [25–27].

#### Polyquad

Polyquad (Alcon) is a hydrophilic cationic quaternary ammonium polymer [80] 27 times larger than BAK [81] with a biocidal mechanism that involves damage to bacterial cytoplasmic membranes causing cell contents to leak out [82]. Its large size and lack of a hydrophobic region may prevent it from entering mammalian cells, thus reducing collateral toxicity to human cells [12]. Polyquad has been used in contact lens solutions [8] (where it does not form deposits on lenses) and in dry eye formulations [6] since the 1980s and more recently has been incorporated into formulations of glaucoma medications. Polyquad is significantly less cytotoxic to the ocular surface compared to BAK [12, 15, 19, 22]. In clinical studies, ocular symptom scores using the OSDI were lower (better) in eyes treated with Polyquad-preserved travoprost than with BAK-preserved travoprost, [53] and eyes switched from the BAK- to Polyquad-preserved formulation manifested improvements in both OSD sign and symptoms [28–30]. As with SofZia, replacing BAK with Polyquad did not affect the IOP-lowering efficacy of glaucoma medication formulations [28–30, 83].

#### Purite

Purite (Allergan, an Abbvie company, Chicago, IL) is a stabilized oxochloro complex (SOC) containing 99.5% chlorite, 0.5% chlorate, and trace chlorine dioxide that converts to sodium and chloride ions, oxygen, and water upon contact with the tear film [7]. Chlorine dioxide free radicals in solution impart Purite’s antimicrobial activity; it is effective against bacteria and viruses [7]. Purite is less cytotoxic to the ocular surface than BAK [84–86]. In clinical studies, brimonidine preserved with Purite was better tolerated than BAK-preserved brimonidine in glaucoma patients with signs and/or symptoms of OSD and delivered comparable IOP reduction [87–89].

### Preservative-free formulations

Select topical ophthalmic therapies for the treatment of glaucoma, OSD, and allergic eye disease, among others, are available in preservative-free formulations (examples given in Table 5). Collectively, as would be expected, these formulations demonstrate less cytotoxicity than their corresponding BAK-preserved formulations [14, 16–18]. In clinical studies, OSD signs and symptoms are consistently better with preservative-free versus preserved formulations [45–48, 50, 51, 90–100]. No compromise in IOP control has been identified with preservative-free versus preserved formulations of glaucoma medications [50, 51, 90, 91, 93, 101]. The concentration of latanoprost free acid in the aqueous humour is above the threshold for prostaglandin receptor F activation following dosing with both BAK-preserved and preservative-free latanoprost [102].

**Table 5:** Representative preservative-free topical formulations available for the treatment of glaucoma, ocular surface disease, and ocular allergy in the United States.

Drug class	Examples of commercial preservative-free formulations
Steroids	None
NSAIDs	Acuvail (Allergan, an AbbVie company), Omidria (Omeros)
Antibiotics	Vigamox (Novartis)
Ocular hypotensives	Zioptan (Oak pharmaceutiicals), Cosopt PF (Merck), Timoptic in Ocudose (Bausch & Lomb)
Immunosuppressants	Restasis (Allergan, an AbbVie company), Cequa (Sun pharma)
Antihistamines	Alaway (Bausch & Lomb)
Hypertonics	Muro 128 (Bausch & Lomb)

Preservative-free formulations have significant limitations to be considered, the most important of which is that many topical therapies are not available in preservative-free formulations, and those that are available are almost without exception more expensive than their preserved counterparts and are subject to limited coverage by most insurers. These products are most commonly packaged as single-dose vials meant to be discarded after each use to prevent microbial contamination. This benefit is often thwarted by patients who save the vials after first use in order to extract subsequent doses before discarding. They are difficult for many patients—particularly older people at highest risk for age-related diseases such as glaucoma and OSD—to use. The vials are typically attached to one another and must be peeled apart. Instead of a screw-cap, they are opened by tearing the top of the plastic vial apart from the drug-containing reservoir, which requires some dexterity and can leave rough and/or sharp edges that can pose the risk of ocular surface injury if unintentional contact occurs between the vial and the eye during dosing. Their small size can make gripping and squeezing difficult, especially in patients with tremor or arthritis of the hands. Some of these obstacles can be overcome with multidose preservative-free formulations, but these are at risk of microbial contamination [103]. Systems to prevent microbial influx into multidose preservative-free formulations—such as the proprietary ABAK and COMOD systems—have been developed and are available for a limited number of drugs [3]. Also, preservative-free liposomal eye sprays have been developed for the treatment of OSD [104].

### Preservative-free sustained drug delivery platforms

Sustained drug delivery describes the administration of a single depot of preservative-free drug that is delivered to the target tissue(s) at the intended therapeutic concentration for a sustained period of time, avoiding the peaks and troughs of pulsed topical dosing. There are numerous advantages and disadvantages to these drug delivery platforms. As for advantages, these options reduce or eliminate the need for patient self-dosing, which in turn reduces or eliminates nonadherence and therefore may be associated with improved clinical outcomes and quality of life. Sustained drug delivery can significantly reduce the treatment burden imposed by daily topical dosing of glaucoma medications or reduce the frequency of intravitreal injections for ocular inflammatory disease, for example. Drugs can be delivered directly to target tissues, which may include the ocular surface to treat OSD, both the ocular surface and the anterior segment to treat glaucoma and postoperative inflammation, and the posterior segment to treat retinal diseases. Intraocular drug delivery spares the ocular surface exposure to active and excipient ingredients. Both steady-state and tapering drug levels can be delivered by sustained drug delivery platforms over time periods ranging from 30 days to three years by altering design elements.

Disadvantages of sustained drug delivery platforms include the need for administration by healthcare professionals in most cases, often via medical or surgical procedures that may incur procedure-related safety events in addition to drug-related side effects, although in some cases administration can be paired with planned surgical procedures to minimize added risk or exposure. Dose titration—strength/concentration, dosing schedule, etc.—is not possible as the daily dose delivered is a function of each platform’s design. For intraocular platforms, it can be difficult or impossible to discontinue therapy before the drug is fully depleted, such that drug-related side effects may be difficult to manage. Existing platforms are designed for single-drug delivery only, which limits the value of these options for patients who require multiple drugs to manage their ocular condition(s). There is the theoretical possibility of reduced efficacy compared to standard pulse dosing, for example in the case of receptor saturation and downregulation with sustained release of prostaglandins for glaucoma therapy [105, 106]. In addition, sustained-release formulations may be more expensive than their non-sustained release counterparts, and the value of these products’ attributes may not be perceived as of sufficient value to payors to cover the added costs.

Sustained release technology is not new to ocular therapeutics. More than 40 years ago the Ocusert was a sustained delivery system that resided on the ocular surface in the conjunctival fornix and delivered pilocarpine for IOP control for a week at a time [107]. In the 1990s, a ganciclovir intravitreal implant was developed for the treatment of cytomegalovirus retinitis [108] before the development of highly active antiretroviral therapy all but eradicated CMV among patients with acquired immunodeficiency syndrome (AIDS). In more recent years, numerous novel sustained drug delivery platforms have been commercialized for a variety of ocular conditions and diseases, and many others are in various stages of preclinical and clinical development [109–112]. Several commercialized sustained drug delivery platforms are described below as examples of this rapidly evolving therapeutic frontier.

#### Implants and Inserts

Durasert (EyePoint Pharmaceuticals, Watertown, MA) is a small, injectable sustained delivery system that can deliver small molecules at therapeutic concentrations for up to three years without the need for preservatives. This technology is based on a permeable polyvinyl alcohol membrane positioned between the drug reservoir and the device’s aperture that controls the rate of drug elution. The platform is nonerodable and a capsule remains in the eye after complete drug elution. The system has been incorporated into several commercial products to treat a variety of chronic ocular conditions [113], including the aforementioned ganciclovir implant and three fluocinolone acetonide intravitreal implants (Retisert 0.59 mg, Bausch & Lomb, Rochester, NY; Iluvien 0.19 mg, Alimera Sciences, Alpharetta, GA; and Yutiq 0.18 mg, EyePoint). The Retisert device is classified as an implant as it is sutured to the inner eyewall via a surgical procedure, while the remaining 2 fluocinolone devices are inserts and can be injected without a surgical procedure.

The NOVADUR system (Allergan, an AbbVie company) is another notable sustained delivery system. In this system, the active drug is embedded in a polymer matrix of poly (D,L-lactide-co-glycolide) (PLGA) that slowly degrades to lactic and glycolic acids which in turn degrade to water and carbon dioxide upon drug depletion, leaving no residue in the eye [113]. Two biodegradable injectable ocular inserts—a dexamethasone intravitreal insert for posterior segment inflammation (Ozurdex, dexamethasone 0.7 mg) and a bimatoprost anterior chamber insert for glaucoma therapy (Durysta, bimatoprost 10 mcg) have been commercialized by the manufacturer (Allergan, Dublin, Ireland) and provide preservative-free drug delivery for ~6 months.

Hydrogels are another promising sustained release drug delivery platform. Hydrogels are biocompatible hydrophilic crosslinked polymer networks that swell when exposed to water [114]. Active drug is incorporated into the polymer matrix without preservatives. The rate of drug delivery, and thus the duration of action of a hydrogel-based therapeutic, is determined by the degree of polymeric crosslinking and the relative sizes of the inter-crosslink mesh openings and the drug to be delivered, the latter of which is fixed while the former of which can be varied in the design and synthesis processes [114, 115]. The DEXTENZA^®^ intracanalicular insert (Ocular Therapeutix, Bedford, MA) is a preservative-free sustained-release formulation of dexamethasone 0.4 mg encapsulated in a hydrogel sustained delivery system indicated for control of inflammation and pain following ophthalmic surgery. As it dissolves within the canaliculus, the insert delivers a tapering dose of dexamethasone to the tear film up to 30 days. Hydrogel-based sustained release therapeutics are also being developed for the treatment of ocular surface diseases and intracameral delivery of travoprost for glaucoma as well as intravitreal delivery of tyrosine kinase inhibitors for retinal vascular disorders [116, 117].

The Verisome platform (EyePoint) is a versatile sustained drug delivery platform that can deliver large or small molecules for periods ranging from weeks to months formulated into biodegradable solids, gels, or liquids [116]. Dexycu (EyePoint) is a 9% suspension of dexamethasone delivered intraocularly at the time of surgery to control postoperative inflammation [116]. Upon injection, it coalesces to form a single spherule that biodegrades over ~21 days, releasing dexamethasone in a tapering dose over time.

### Non-Pharmacological Therapy

At the extreme end of BAK avoidance is the eschewing of medication therapy altogether. While medication therapy is the preferred first-line approach for glaucoma treatment, laser therapy (selective laser trabeculoplasty) [118], minimally invasive glaucoma surgeries [119–122], and even primary filtering surgery [123, 124] can be considered alternatives to medication therapy. For the management of OSD, punctal plugs, intranasal neurostimulation, and Meibomian heat therapy are all non-medication options [125]. Further, for allergic eye disease, allergen immunotherapy is an alternative to topical medical therapy [126].

## Unmet need and future developments

BAK is nearly ubiquitous in topical ophthalmic formulations and is associated with multiple adverse effects on ocular health that are aggravated with chronic exposure. As discussed above, there are many options for alternate therapies to treat a variety of conditions in patients for whom BAK avoidance is desired or necessary. Many of these, however, have limitations, including expense, availability, invasiveness, and a reduction or elimination of dose titration. There remains a significant unmet need for further development of widely available, and easy-to-administer BAK-free therapies for common ocular conditions. Innovation in bottle design might reduce preservative exposure at the point of dosing [66, 127, 128]. Novel excipients could be added to BAK-preserved formulations to diminish adverse effects without altering biocidal activity [129]. Alternate preservatives that are less damaging to the ocular surface would have value [130], and expanding the range of commonly-used medications available in preservative-free formulations would make a broad impact as well, particularly if packaging can be redesigned to optimize user-friendliness. Of particular impact would be the expansion of sustained drug delivery platforms and products to provide preservative-free therapeutic options that eliminate the need for patient adherence. These would have the greatest impact in chronic conditions where reductions in treatment burden can contribute to improved quality of life. Most importantly, the prioritization of preservative-free therapy to spare the ocular surface will require recognition and valuation by health payors to ensure that patients have affordable access to existing and emerging therapeutic options.
